# Disparities in the access to atrial fibrillation ablation in Denmark: who gets ablated, who neglected?

**DOI:** 10.1093/europace/euae231

**Published:** 2024-09-04

**Authors:** Christopher R Zörner, Jacob Tønnesen, Lise Da Riis-Vestergaard, Charlotte Middelfart, Regitze Hein, Peter Vibe Rasmussen, Martin H Ruwald, Gunnar Gislason, Morten Lock Hansen

**Affiliations:** Department of Cardiology, Herlev-Gentofte University Hospital, University of Copenhagen, Gentofte Hospitalsvej 6, 2900 Hellerup, Denmark; Department of Cardiology, Herlev-Gentofte University Hospital, University of Copenhagen, Gentofte Hospitalsvej 6, 2900 Hellerup, Denmark; Department of Cardiology, Herlev-Gentofte University Hospital, University of Copenhagen, Gentofte Hospitalsvej 6, 2900 Hellerup, Denmark; Department of Cardiology, Herlev-Gentofte University Hospital, University of Copenhagen, Gentofte Hospitalsvej 6, 2900 Hellerup, Denmark; Department of Cardiology, Herlev-Gentofte University Hospital, University of Copenhagen, Gentofte Hospitalsvej 6, 2900 Hellerup, Denmark; Department of Cardiology, Herlev-Gentofte University Hospital, University of Copenhagen, Gentofte Hospitalsvej 6, 2900 Hellerup, Denmark; Department of Cardiology, Herlev-Gentofte University Hospital, University of Copenhagen, Gentofte Hospitalsvej 6, 2900 Hellerup, Denmark; Department of Cardiology, Herlev-Gentofte University Hospital, University of Copenhagen, Gentofte Hospitalsvej 6, 2900 Hellerup, Denmark; The Danish Heart Foundation, Copenhagen, Denmark; Department of Clinical Medicine, Faculty of Health and Medical Sciences, University of Copenhagen, Copenhagen, Denmark; The National Institute of Public Health, University of Southern Denmark, Copenhagen, Denmark; Department of Cardiology, Herlev-Gentofte University Hospital, University of Copenhagen, Gentofte Hospitalsvej 6, 2900 Hellerup, Denmark; Department of Clinical Medicine, Faculty of Health and Medical Sciences, University of Copenhagen, Copenhagen, Denmark

**Keywords:** Atrial fibrillation, Ablation, Disparity, Denmark, Healthcare access

## Abstract

**Aims:**

Atrial fibrillation (AF) is a common arrhythmia associated with reduced quality of life that can lead to serious complications such as stroke and heart failure. Ablation is a safe and effective treatment for AF but is not offered equally to all patients. The aim of this study is to identify demographic groups more or less likely to undergo AF ablation.

**Methods and results:**

All patients with newly diagnosed AF between 2010 and 2018 were identified in the Danish nationwide registries. The association between gender, age, level of education and attachment to the job market, and the likelihood of receiving AF ablation was investigated using multivariable Cox proportional hazard analysis. Cumulative incidence was calculated using the Aalen–Johansen estimator. A total of 176 248 patients were included. Men were more likely to receive ablation than women (7% vs. 3%). Patients aged 25–44 and 45–64 were most likely to receive ablation, while only 0.7% of patients aged 80 or above received ablation. The rate of ablation significantly decreased with decreasing level of education. Full-time employed patients were most likely to receive ablation, followed by self-employed, unemployed, on sick leave, undergoing education, and early retired patients. Retired patients were the least likely to receive ablation (3%).

**Conclusion:**

This study found that women, older patients, patients with lower levels of education, and patients on social benefits are less likely to receive AF ablation. These findings suggest that there are significant social and economic disparities in AF ablation treatment in Denmark.

## Graphical Abstract

**Graphical Abstract:**
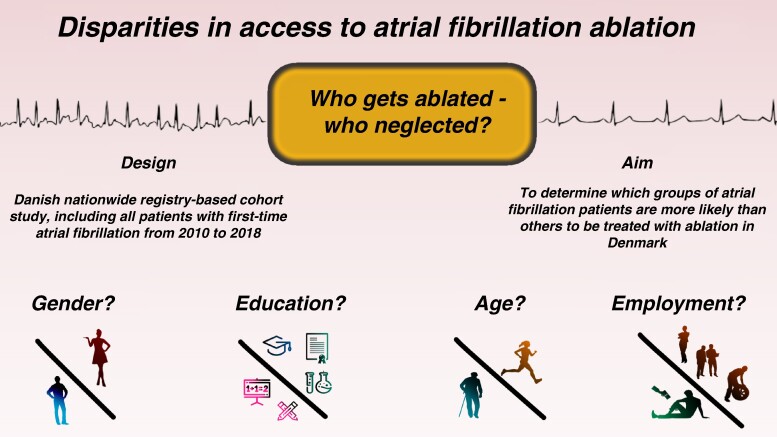

**What’s new?**

- Socioeconomic factors: Education level, employment status, and other socioeconomic factors were shown to significantly influence the likelihood of receiving atrial fibrillation (AF) ablation, with higher education being associated with increased rates of ablation.
- Age-related trends: Older age groups were less likely to receive AF ablation, highlighting a potential age bias in clinical decision-making despite the increasing prevalence of AF with age.
- Gender disparities: The analysis revealed gender disparities in AF ablation rates, with women being less likely to undergo the procedure compared to men, suggesting potential gender-based differences in treatment approaches.
- Impact of comorbidities on ablation likelihood: The study identifies that patients with ischaemic heart disease, chronic kidney disease, and other significant comorbidities were less likely to undergo AF ablation, suggesting potential hesitancy in offering ablation to more complex patients. Notably, however, patients with prevalent heart failure were more likely to undergo AF ablation.

## Introduction

Atrial fibrillation (AF) is the most common arrhythmia worldwide and associated with increased risk of cardiovascular and thrombotic complications.^1,2^ Other than the well-established association between AF and cardiovascular morbidity and mortality, the presence of symptomatic AF has been shown to affect patients perceived quality of life (QoL), mental health, and cognitive function.^3–9^

Radiofrequency ablation and cryoballoon ablation have been proven as safe, viable, and effective treatment options for patients with AF and are increasingly being offered as first-line therapy.^10–12^

Despite significant advances in the field of ablation and the increasingly prominent role of the procedure within AF treatment regional disparities in availability, patient profile and therapeutic strategy remain prevalent.^13^

Factors such as gender, income, and educational level have been described to influence therapeutic decision-making within cardiovascular disease.^14,15^ Several studies examining these disparities have highlighted the negative effects on outcome and QoL these decisions can have.^16–18^

Given the importance of optimal AF treatment and the prominence of AF ablation in international guidelines and clinical practise, examinations into the factors influencing the clinical decision-making process, of who to ablate and at what time, are warranted. Therefore, this study was designed to determine which groups of AF patients were more likely than others to be treated with AF ablation in Denmark.

## Methods

### Study population and design

Thais study was designed as a nationwide registry-based cohort study. All Danish patients of 18 years or more, diagnosed with first-time AF (International Classification of Disease, 10th revision (ICD-10): I48) between 1 January 2010 and 31 December 2018, both inpatient and outpatient, were included at the time of diagnosis.

### Outcome of interest

Atrial fibrillation ablation was defined by procedure codes BFF03 and BFF04.

### Data sources

This study was based on several nationwide registers: The Danish National Patient Register,^19^ The Civil Registration System,^20^ The Danish Registry of Medicinal Product Statistics,^21^ and databases provided by Statistic Denmark on employment status and level of education, more specifically the module on labour market classification^22^ and the module on the classification of education. These nationwide registers were cross-linked on the individual level using the unique permanent identification number given to all Danish residents at birth or migration.

### Variables

Variables analysed were gender, patient age, level of education, employment status, and prevalence of common comorbidities. For the purpose of comparative analysis, patients were divided into one of groups by age: age 24 years or younger, age 25–44, age 45–64, age 65–79, and 80 years or above. Likewise patient level of education was classified by the highest level of education achieved by time of inclusion. These categories were as follows: basic school education, high school/vocational training, and non-university higher education; university: bachelor’s degree; university: master’s degree; and university: PhD/Doctorate. Patients without registered level of education were classed as unknown/other.

To evaluate the impact of attachment to the job market, patients were classed in accordance with their level of employment at time of AF diagnosis. Patients could be classed as employed, self-employed, undergoing education, unemployed, on sick leave, early retired, and retired. Patients without registered level of employment were classed as unknown/other.

Comorbidities analysed included heart failure, ischaemic heart disease (IHD), ischaemic stroke, chronic kidney disease (CKD), hypertension, and chronic obstructive pulmonary disease and were registered 5 years before study inclusion. ICD-10 codes used for these comorbidities are provided in Supplementary material online, *Table S1*.

Concomitant pharmacotherapy at baseline was defined as any claimed prescription 180 days prior to the date of study inclusion. Medications included in the analysis were beta-blockers, calcium channel blockers, renin–angiotensin system inhibitors, loop diuretics, spironolactone, oral anticoagulant therapy, digoxin, and amiodarone. Oral anticoagulant comprised warfarin, phenprocoumon, dabigatran, rivaroxaban, apixaban, and edoxaban. ATC codes used for these pharmaceuticals are provided in Supplementary material online, *Table S2*.

### Statistical analysis

Descriptive tables were employed to describe the study population and comorbidities, level of education and employment with continuous variables reported as medians and interquartile ranges [IQRs], and categorical variables summarized with counts and corresponding percentages. The cumulative incidence of AF ablation was calculated utilizing the Aalen–Johansen estimator and death accounted for as a competing risk factor. Multivariable Cox proportional hazard analysis was used to examine the association between gender, age group, level of employment and education, and comorbidities in newly diagnosed AF patients and their likelihood of undergoing AF ablation. Results are reported in form of hazard ratios (HRs) with 95% confidence interval (CI). A two-sided *P*-value of <0.05 was considered significant.

Statistical analysis and programming were conducted using R statistical software.

### Ethics

According to Danish legislation, retrospective studies using administrative health databases do not require ethical approval in Denmark. The Danish Data Protection Agency has approved the use of registry data, and the current project is registered (approval number: P-2019-408).

## Results

### Demographics

During the observation period, a total of 176 248 patients with newly diagnosed AF were identified and included in the study. Median age at time of inclusion was 74 years (IQR 66, 82), and 79 222 (45%) of the cohort were women.

In term of education, 40% of patients in the cohort had only finished basic school education and 36% had finished high school or vocational training. A bachelor’s degree was attained by 11% of patients and master’s degree by 5%, and <1% had finished a PhD or doctorate degree. Further 3% were classified as having a non-university higher education, and a further 6% had an education status that was either unknown or otherwise not characterized.

Most patients were retired at time on inclusion (76%), while 15% were full time employed, 2% classed as self-employed and 4% early retired. Less than 1% of patients included were either registered as being on sick leave, undergoing education or as unemployed, while for 2% of the cohort, their employment status was unknown.

All these observations are summarized in *Table 1*.

**Table 1:** Baseline characteristics

	All	Ablated	Not ablated
Number of patients	176.248	8.382	167.866
Age (median [IQR])	74 [66, 82]	61 [53, 67]	73 [67, 82]
Age categories by years: (%)			
24 or below	14.027 (8)	894 (11)	13.133 (8)
25–44	3.971 (2)	549 (6)	3.422 (2)
45–64	30.356 (17)	4.236 (50)	26.120 (16)
65–79	70.496 (40)	2.568 (31)	67.928 (40)
80 or above	57.398 (33)	137 (2)	57.263 (34)
Gender: women (%)	79.222 (45)	2.254 (27)	76.968 (46)
Education (%)			
Basic school education	70.624 (40)	1.782 (21)	68.842 (41)
High school/vocational training	63.231 (36)	3.759 (45)	59.472 (35)
Non university higher education	4.922 (3)	424 (5)	4.498 (3)
University: bachelor	19.275 (11)	1.375 (16)	17.900 (11)
University: master	7.991 (5)	763 (9)	7.228 (4)
University: PhD/doctorate	292 (<1)	55 (<1)	237 (<1)
Unknown/other	9.913 (6)	224 (3)	9.689 (6)
Employment (%)			
Employed	25.686 (15)	4.009 (48)	21.677 (13)
Early retired	6.681 (4)	394 (5)	6.287 (4)
Retired	13.4612 (76)	3.018 (36)	13.1594 (78)
Self-employed	3.955 (2)	472 (6)	3.483 (2)
Sick leave	801 (<1)	92 (1)	709 (<1)
Undergoing education	435 (<1)	35 (<1)	400 (<1)
Unemployed	537 (<1)	63 (<1)	474 (<1)
Unknown/other	3.541 (2)	299 (4)	3.242 (2)
Comorbidities (%)			
Ischaemic heart disease	30.880 (18)	1.046 (12)	29.834 (18)
Hypertension	86.067 (49)	2.699 (32)	83.368 (50)
Heart failure	23.848 (14)	917 (11)	22.913 (14)
Ischaemic stroke	19.866 (11)	308 (4)	19.558 (12)
Chronic obstructive pulmonary disease	19.247 (11)	280 (3)	18.967 (11)
Chronic kidney disease	9.508 (5)	137 (2)	9.370 (6)
Pharmacotherapy (%)			
RAS inhibitors	72.324 (41)	2.581 (31)	69.743 (41)
Beta-blockers	63.584 (36)	3.120 (37)	60.464 (36)
Calcium channel antagonists	45.101 (26)	1.365 (16)	43.736 (26)
Loop diuretics	36.759 (21)	705 (8)	36.054 (21)
Amiodarone	1.346 (<1)	75 (<1)	1.271 (<1)
Digoxin	9.708 (6)	263 (3)	9.445 (6)
Oral anticoagulants	43.820 (25)	2.359 (28)	41.461 (25)
Spironolactone	10.169 (6)	276 (3)	9.893 (6)

IQR, interquartile range; RAS, renin–angiotensin system.

### Atrial fibrillation ablation by gender

**Figure 1:**
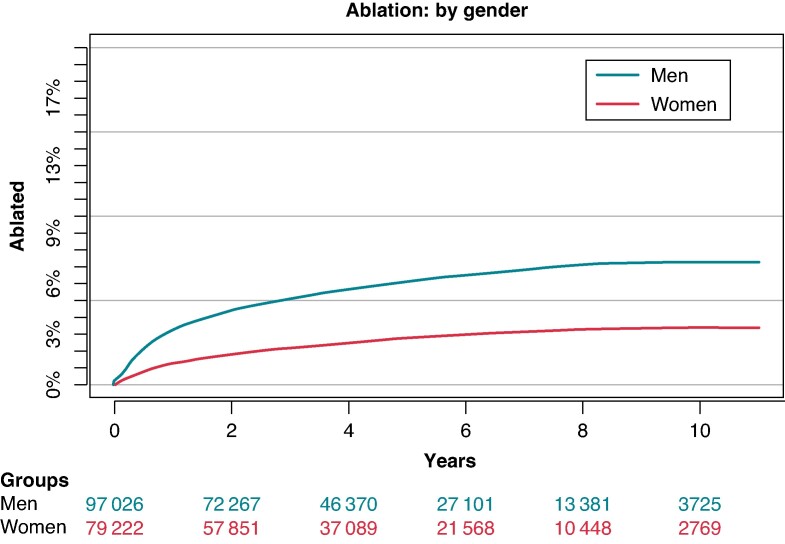
Cumulative incidence of AF ablation: divided by gender. AF, atrial fibrillation.

Analysing the rate of patients who went on to receive AF ablation revealed a striking disparity between genders. Over the observation period, 7% of men were ablated, while this was only the case for 3% of women (*Figure 1*).

**Figure 2:**
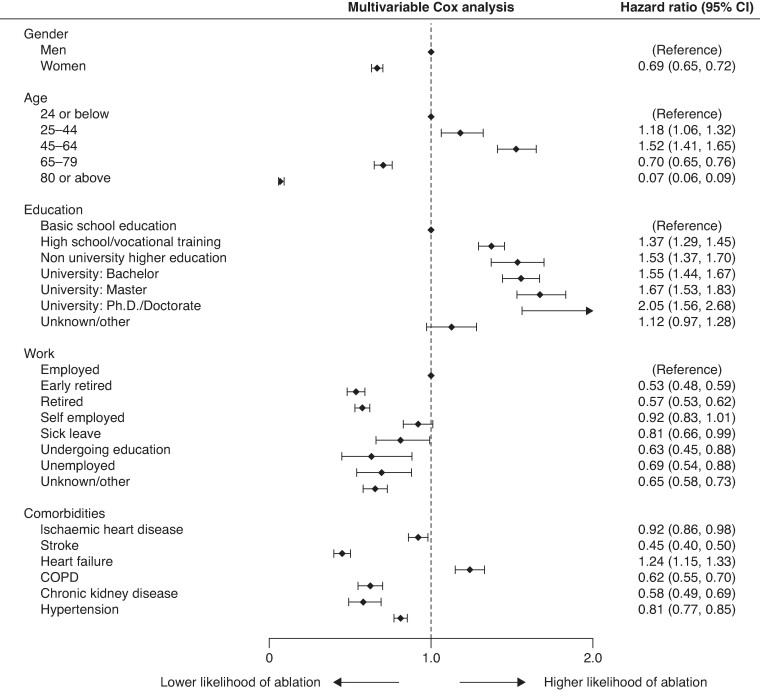
Forest plot with HR for AF ablation. AF, atrial fibrillation; CI, confidence interval; HR, hazard ratio.

In multivariable Cox proportional hazard analysis, the HR for women for receiving ablation was valued at 0.69 [95% CI (0.65–0.72)], indicating a significantly lower likelihood of women to be treated with AF ablation compared with men (*Figure 2*).

### Atrial fibrillation ablation by age

**Figure 3:**
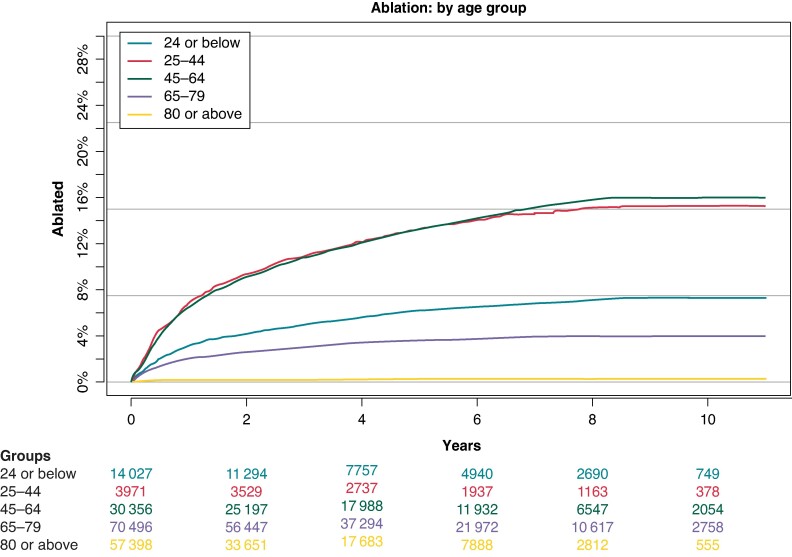
Cumulative incidence of AF ablation: divided by age group. AF, atrial fibrillation.

This analysis showed a clear trend of patients aged 25–44 and 45–64 being most frequently offered AF ablation, with 16% and 17% of these groups respectively undergoing the procedure. At a rate of 8%, patients aged 24 or lower were offered ablation, while this was the case for 4% of patients aged 65–79. The lowest rate was seen in those aged 80 or above with <1% being ablated for AF (*Figure 3*).

These findings were also reflected in multivariable Cox proportional hazard analysis. With patients aged 24 or below were taken as a reference group. Those aged 25–44 had a HR of receiving ablation of 1.18 [95% CI (1.06–1.32)] while this was 1.52 [95% CI (1.41–1.65)] for patients aged 45–64 years. Lower HRs were observed for patients between 65 and 79 years with 0.70 [95% CI (0.65–0.76)] as well as for those aged 80 or above with 0.07 [95% CI (0.06–0.09)] (*Figure 2*).

### Atrial fibrillation ablation by level of education

**Figure 4:**
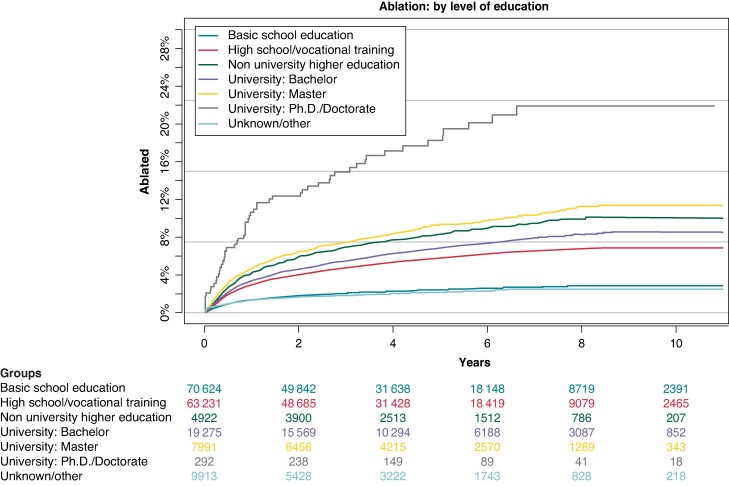
Cumulative incidence of AF ablation: divided by level of education. AF, atrial fibrillation.

Level of education was another factor that revealed significant disparities between groups. The lowest rates of AF ablation were observed among patients who had only received basic school education as well as those with an unknown or classed other level of education at around 2% each. Increasingly higher rates were seen for those who had finished high school or vocational training at 7%, while 9% of patients with a university bachelor’s degree were ablated and 10% with a non-university higher education. Of those patients with a complete master’s degree, 11% were ablated while by far the highest rate was observed among patients with a PhD or doctorate title with 22% in this group receiving AF ablation (*Figure 4*).

In Cox proportional hazard analysis, patients with only basic school education were taken as a reference group. This analysis revealed increasing HR with increasing level of education, starting with 1.37 [95% CI (1.29–1.45)] for patients with finished high school education or vocational training, 1.53 [95% CI (1.37–1.70)] for those with a non-university higher education, 1.55 [95% CI (1.44–1.67)] for patients with a university bachelor’s degree, 1.67 [95% CI (1.53–1.83)] with a master’s degree, and finally 2.05 [95% CI (1.56–2.68)] for patients with a finished PhD or doctorate. Patients classified as unknown or other had a HR of 1.12 [95% CI (0.97–1.28)] for receiving ablation in this analysis (*Figure 2*).

### Atrial fibrillation ablation by employment status/attachment to the job market

**Figure 5:**
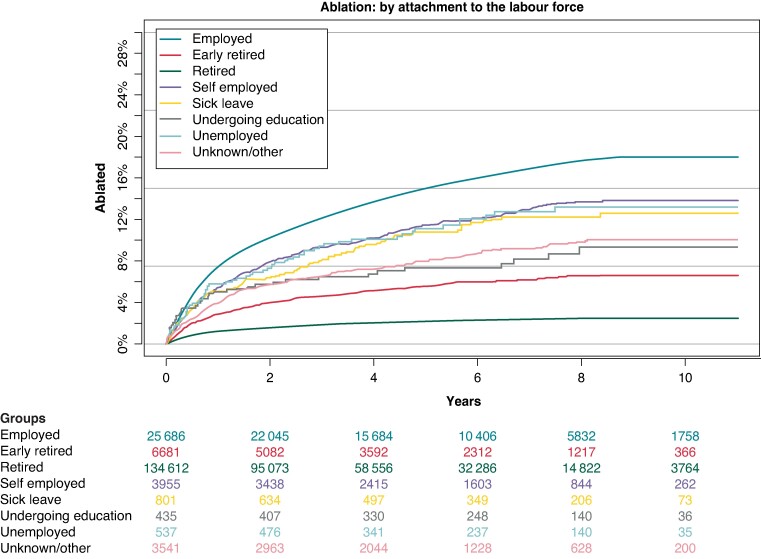
Cumulative incidence of AF ablation: divided by employment status. AF, atrial fibrillation.

Fully employed patients were most likely to receive AF ablation with 18% in their group. Thereafter, patients classed as self-employed, unemployed, and on sick leave had comparable rates of ablation at between 12 and 14%. Following these groups, patients undergoing education and those with an unknown status or classified as other were ablated at a rate of 10% and 9%, respectively, while this was the case for around 7% of patients in early retirement. Lastly, retired patients were ablated at a rate of 3% (*Figure 5*).

In the Cox proportional hazard analysis, patients who were full time employed were used as reference group. Patients in early retirement were less likely to receive AF ablation with a HR of 0.57 [95% CI (0.48–0.59)] as were those in full retirement [HR 0.57; 95% CI (0.53–0.62)]. Similarly, rates were lower for patients undergoing education with a HR of 0.63 [95% CI (0.45–0.88)], those registered as unemployed with a HR of 0.69 [95% CI (0.54–0.88)] as well as patients with an unknown status or classed otherwise [HR 0.65; 95% CI (0.58–0.73)]. Lower but statistically insignificant rates were calculated for self-employed patients [HR 0.92; 95% CI (0.83–1.01)], while only the HR for patients on sick leave was only marginally significant with HR 0.81 [95% CI (0.66–0.99)] (*Figure 2*).

### Comorbidity

Finally, the prevalence of common comorbidities was analysed for its impact on the likelihood of AF ablation. This analysis showed lower rates of ablation among patients with prior history of IHD [HR 0.92; 95% CI (0.86–0.98)], ischaemic stroke [HR 0.45; 95% CI (0.4–0.5)], chronic obstructive pulmonary disease [HR 0.62; 95% CI (0.55–0.7)], CKD [HR 0.58; 95% CI (0.49–0.69)], and hypertension [HR 0.81; 95% CI (0.77–0.85)]. Patients with a history of heart failure were conversely more likely to undergo AF ablation with a HR of 1.24 [95% CI (1.15–1.33)] (*Figure 2*).

## Discussion

The present study presents a comprehensive analysis of demographic factors influencing the likelihood of undergoing AF ablation in Denmark, utilizing nationwide registries spanning from 2010 to 2018. The findings reveal significant disparities, shedding light on the social and economic dimensions affecting the access and utilization of AF ablation treatments. The findings revealed significant disparities in ablation rates among different demographic groups. Women, older patients, patients with lower levels of education, and patients who were not employed or retired were less likely to receive ablation. These findings suggest that there are social and economic factors that may contribute to these disparities.

### Gender disparities

One striking observation is the substantial gender gap in AF ablation rates, with men being almost twice as likely to undergo the procedure compared with women. Gender-based differences and biases are well described within cardiovascular care. In the context of AF, existing studies have shown that women are generally older at time of AF diagnosis and more symptomatic.^23,24^ Furthermore, women have been shown to be less likely to receive AF ablation than men. Much effort has been made to explain this disparity exploring several possible explanations. The most common explanation suggests that AF ablation is less effective in women and complication rates, especially thromboembolic events, are higher.^25–27^ However, this notion is challenged by other researchers, who reason the observed differences are not a direct result of gender alone, but by differences in age, severity of symptoms as well as longer time to diagnosis and treatment observed among women with AF.^28,29^

### Age-related disparities

The age-stratified analysis demonstrates a clear inverse relationship between age and the likelihood of AF ablation. Younger patients, particularly those aged 25–44 and 45–64, exhibit higher rates of ablation, while the elderly, especially those aged 80 and above, are significantly less likely to undergo the procedure. However, more evidence is emerging that also the elderly can profit greatly from AF ablation and recommend a more extensive inclusion of this procedure in this patient group. Several studies now support the safety and efficacy of ablation in the elderly and highlight the benefits to symptom control and QoL.^30–32^

### Educational disparities

The study underscores a notable association between educational attainment and the likelihood of AF ablation. Patients with higher levels of education, particularly those with PhD or doctorate degrees, are markedly more likely to receive ablation. In contrast, patients with only basic school education have significantly lower rates.

The association between education and AF ablation suggests disparities in factors such as health literacy, access to information, or patient advocacy. Health literacy and access to information have been shown to greatly influence the outcome and choice of therapy patients receive.^33–36^ Overcoming these gaps in information and knowledge will be detrimental in ensuring equal access to healthcare services in the general population.

### Employment status impact

The analysis of employment status reveals intriguing patterns, with full-time employed individuals having the highest likelihood of undergoing AF ablation, even within a universal and publicly funded healthcare system such as in Denmark.

Why patients undergoing education or self-employed displayed lower rates of AF ablation seems challenging to interpret. A possible explanation could be a reluctance of these patients to pause their ongoing education or self-employment to undergo a planned invasive procedure requiring admission, outpatient consultation, and the possibility of procedure-related complications. Faster acting rate control therapies might be preferred and implemented accordingly. To determine whether this or other factors contribute to the observed lower rates of AF ablation among these patients further and more nuances research will be required.

### Comorbidity influence

The study's examination of comorbidities further enriches our understanding of factors influencing AF ablation. Patients with a history of heart failure are more likely to undergo ablation, while those with ischaemic stroke, chronic obstructive pulmonary disease, CKD, and hypertension demonstrate lower ablation rates.

Excluding patients with higher prevalence of comorbidities from invasive procedures such as AF ablation seems plausible, given the higher risk of procedure-related complication. Additionally, recurrence rates have been shown to be higher among patients with higher rates of cardiovascular comorbidity.^37,38^ The decision to pursue AF ablation in heart failure patients might be influenced by growing evidence and clinical experience suggesting that AF ablation can lead to significant improvements in QoL post-procedure. Clinicians, therefore, may be more inclined to recommend ablation to heart failure patients, anticipating these potential benefits. With the advances and potential advantages of AF ablation as a treatment, in more recent years, studies have examined the query of risk and benefit of AF ablation among patients with higher cardiovascular morbidity burden. Trials such as CASTLE-AF, AATAC, and CABANA have highlighted the benefit of ablation in clinical outcome and QoL compared with drug treatment.^39–41^ This increasing evidence has prompted the European Society of Cardiology to recommend catheter ablation for symptomatic patients with heart failure with a IIa recommendation and evidence level B in the current guidelines for the treatment of heart failure from 2021.^42^ Similarly, there has been movement towards offering AF ablation as a treatment option to patients with ever-increasing morbidity burdens, as reflected in such developments in international guidelines.

### Strengths and limitations

The strengths of this study include the large sample size, the use of nationwide registry data, and the long follow-up period. The Danish National Patient Registry and the general completeness of data in the Danish nationwide registries ensure minimal missing data and a complete follow-up.^43^

While this study provides valuable insights, certain limitations should be acknowledged. The retrospective nature of the study using registry data might introduce biases, and the specific reasons behind the observed disparities require further qualitative exploration. Additionally, the study focuses on the Danish population, and generalizability to other healthcare systems and populations may vary. A further strength is the real-world experience nature of the data and the resulting possibility to correlate to compliance of guidelines and identify potential for better dissemination and use.

The analysis could not differentiate between various subtypes of AF, such as paroxysmal, persistent, and permanent AF, due to the lack of data in the national registries. This limitation prevents a detailed evaluation of how the type of AF might influence ablation decisions and outcomes.

Additionally, the data utilized in this study pertain to the period from 2010 to 2018. Given that the European Society of Cardiology guidelines on AF management were updated in 2016 and 2020 and that the prevalence of AF ablation has increased over time, the results may not fully reflect current treatment practices and guidelines. Likewise procedural advances within the field of AF ablation during the analysed perode likely influenced the availability and patient selection.^44^ In order to reflect possible changes over time, ablation rates for each analysed category were divided into three periods of 2010–12, 2013–15, and 2016–18 have been made available in Supplementary material online, *Table S3*. The data reveal subtle changes over time, as well as few narrowing gaps, e.g. increasing rate of women and older patients ablated. However, despite these trends, the overall distinct disparities of the main analysis overwhelmingly remain unchanged.

Factors such as education level, employment status, age, and gender may exhibit covariant relationships that could influence the study’s findings. For example, lower educational attainment may correlate with a higher likelihood of unemployment or early retirement, and age can negatively correlate with employment status. Furthermore, the distribution of AF patients by gender varies across age groups, with younger individuals generally having higher education levels due to increased educational opportunities over time. These covariant relationships were not fully explored in our analysis, which may have impacted the interpretation of individual factors. Future research should consider the complex interplay between these variables to provide a more nuanced understanding of their effects on AF ablation rates

Furthermore, the study did not include data on left ventricular ejection fraction (EF), which is a critical parameter in assessing the benefit of AF ablation, especially in patients with heart failure with reduced EF. The absence of EF data limits our ability to analyse its impact on ablation decisions and outcomes.

Despite these limitations, the findings provide valuable insights into the demographic and socioeconomic factors influencing AF ablation. Future research incorporating these additional parameters and utilizing more recent data could offer a more comprehensive understanding of the evolving landscape of AF treatment.

## Conclusion

The identified disparities in AF ablation rates underscore the need for a more comprehensive and equitable approach to cardiovascular care. Additionally, further research is warranted to explore the underlying factors contributing to these disparities, including healthcare provider biases, patient preferences, and systemic barriers.

By addressing these challenges, we can work towards a healthcare system that delivers optimal and unbiased care to all individuals diagnosed with AF.

## Clinical perspectives

### Gender disparities

This study reveals a substantial gender gap in AF ablation rates, with men being almost twice as likely to undergo the procedure compared with women. Addressing this disparity is crucial for ensuring equitable access to cardiovascular care and improving outcomes for all patients with AF.

### Age-related considerations

The inverse relationship between age and the likelihood of AF ablation highlights the need for tailored treatment approaches for elderly patients. Despite historically lower rates of ablation among the elderly, emerging evidence suggests potential benefits in symptom control and QoL, warranting further evaluation and consideration in clinical practice.

### Educational attainment and health literacy

Patients with higher levels of education exhibit markedly higher rates of AF ablation, suggesting disparities in health literacy and access to information. Interventions aimed at improving patient education and empowering individuals with comprehensive knowledge about treatment options are essential for promoting informed decision-making and reducing disparities in care.

### Comorbidity burden and personalized treatment strategies

Our findings underscore the importance of personalized treatment strategies for patients with AF and multiple cardiovascular comorbidities. While certain comorbidities may influence the likelihood of AF ablation, shared decision-making processes between clinicians and patients should consider both the benefits and risks of invasive procedures, ensuring that treatment plans align with individual patient needs and preferences.

## Supplementary Material

euae231_Supplementary_Data

## Data Availability

Data for this study are derived from and accessed through Statistics Denmark. By law, these data are not allowed to be shared. For this reason, data cannot be made available to other researchers.
