# Supplementary material for: Disparities in the access to atrial fibrillation ablation in Denmark: who gets ablated, who neglected?
Source: Europace. 2024 Sep 4;26(9):euae231. doi: 10.1093/europace/euae231 (PMC11411207; doi:10.1093/europace/euae231)
Supplement: euae231_Supplementary_Data [file euae231_supplementary_data.docx]

**Supplementary appendix:**

*Supplementary table 1: Diagnostic and procedural codes used to identify comorbidity*

*Supplementary table 2: ATC-codes used to define medical therapy*

*Supplementart table 3: Changes in ablation rates over periods 2010-2012,* ***2013-2015 and 2016-2018***

**Supplementary table 1**

Diagnostic and procedural codes used to identify comorbidity and procedures.

| Comorbidity and procedures |  |
| --- | --- |
| Atrial fibrillation / Atrial flutter | *ICD-10: I48* |
| Chronic Obstructive Pulmonary Disease | *ICD-10: J42-J44* |
| Ischemic heart disease | *ICD-10: I20-I25* |
| Hypertension | *Treatment with more than one anti-hypertensive medication.* |
| Congestive heart failure | *ICD-10: I50* |
| Ischemic stroke / TIA / systemic embolism | *ICD-10: I63, I64, I74, G458, G459* |
| Chronic Kidney Disease | *ICD-10: N02-N08, N11-N14, N18, N19, N26, N158-N160, N 162, N163, N164, N168, Q61, E102, E112, E132, E142, I120, M321B* |
| Procedure: Atrial fibrillation ablation | *BFFB03, BFFB04* |

**Supplementary table 2:** ATC-codes used to define medical therapy

| Oral anticoagulatnt treatment  (Warfarin, phenprocoumon, dabigatran, rivaroxaban, apixaban, edoxaban) | *ATC-codes:*  *B01AA03, B01AA04, B01AE07, B01AF01, B01AF02, B01AF03* |
| --- | --- |
| Beta-blockers | *C07A, C07B, C07C, C07D, C07F* |
| Calcium channel antagonist | *C08C, C08D, C08E, C08G, C09BB, CO9DB* |
| Spironolactone | *C03D, C03E, C03EB* |
| Loop diuretics | *C03C, C03EB* |
| RAS inhibitors | *C09A, C09B, C09C, C09D,* |
| Digoxin | *C01AA* |
| Amiodarone | *C02BD01* |

**Supplementart table 3: Changes in ablation rates over periods 2010-2012, 2013-2015 and 2016-2018**

| ****Category: Years**** | ****2010-2012**** | ****2013-2015**** | ****2016-2018**** |
| --- | --- | --- | --- |
| **Patients ablated / All AF patients (%)** | 2912 / 54424 (5.4) | 3117 / 60252 (5.2) | 2353 / 61572 (3.8) |
| **Age (median [IQR])** | 60.30 [52.60, 66.40] | 62.00 [54.20, 68.40] | 61.90 [53.90, 69.00] |
| **Gender (%)** |  |  |  |
| Women | 770 (26.4) | 835 (26.8) | 649 (27.6) |
| **Age Category (%)** |  |  |  |
| 24 or below | 346 (11.9) | 324 (10.4) | 224 (9.5) |
| 25-44 | 228 (7.8) | 195 (6.3) | 126 (5.4) |
| 45-64 | 1549 (53.2) | 1493 (47.9) | 1194 (50.7) |
| 65-79 | 745 (25.6) | 1043 (33.4) | 780 (33.1) |
| 80 or above | 44 (1.5) | 62 (2.0) | 29 (1.2) |
| **Comorbidities (%)** |  |  |  |
| Ischemic heart disease | 386 (13.3) | 408 (13.1) | 252 (10.7) |
| Chronic obstructive pulmonary disease | 99 (3.4) | 108 (3.5) | 73 (3.1) |
| Chronic kidney disease | 48 (1.6) | 48 (1.5) | 41 (1.7) |
| Hypertension | 988 (33.9) | 990 (31.8) | 721 (30.6) |
| Heart failure | 305 (10.5) | 358 (11.5) | 254 (10.8) |
| Ischemic stroke | 103 (3.5) | 106 (3.4) | 57 (2.4) |
| **Work Status (%)** |  |  |  |
| Early Retired | 152 (5.2) | 156 (5.0) | 86 (3.7) |
| Employed | 1456 (50.0) | 1420 (45.6) | 1133 (48.2) |
| Retired | 962 (33.0) | 1204 (38.6) | 852 (36.2) |
| Self-employed | 173 (5.9) | 171 (5.5) | 128 (5.4) |
| Sick leave | 37 (1.3) | 34 (1.1) | 21 (0.9) |
| Undergoing Education | 14 (0.5) | 15 (0.5) | 6 (0.3) |
| Unemployed | 27 (0.9) | 18 (0.6) | 18 (0.8) |
| Unknown / Other | 91 (3.1) | 99 (3.2) | 109 (4.6) |
| **Education (%)** |  |  |  |
| Basic School Education | 620 (21.3) | 689 (22.1) | 473 (20.1) |
| High School / Vocational Training | 1297 (44.5) | 1390 (44.6) | 1072 (45.6) |
| Non-University Higher Education | 153 (5.3) | 160 (5.1) | 111 (4.7) |
| University: Bachelor | 498 (17.1) | 502 (16.1) | 375 (15.9) |
| University: Master | 259 (8.9) | 272 (8.7) | 232 (9.9) |
| University: Ph.D. / Doctorate | 14 (0.5) | 23 (0.7) | 18 (0.8) |
| Unknown / Other | 71 (2.4) | 81 (2.6) | 72 (3.1) |
